# Impact of Light-Chain Variants on the Expression of Therapeutic Monoclonal Antibodies in HEK293 and CHO Cells

**DOI:** 10.3390/antib14030053

**Published:** 2025-06-24

**Authors:** Alexander Veber, Dennis Lenau, Polyniki Gkragkopoulou, David Kornblüh Bauer, Ingo Focken, Wulf Dirk Leuschner, Christian Beil, Sandra Weil, Ercole Rao, Thomas Langer

**Affiliations:** 1 Sanofi-Aventis Deutschland GmbH, R&D Large Molecules Research, Industriepark Höchst, 65926 Frankfurt am Main, Germany; alexander.veber@sanofi.com (A.V.);; 2 Department of Biopharmaceutical Science, Provadis School of International Management and Technology AG, Industriepark Höchst, 65926 Frankfurt am Main, Germany

**Keywords:** Immunoglobulin G (IgG), Immunoglobulin M (IgM), Kappa and Lambda light chain, recombinant expression, HEK cells, CHO cells

## Abstract

Recombinantly produced monoclonal antibodies (mabs) belong to the fastest growing class of biotherapeutics. In humans, antibodies are classified into five different classes: IgA, IgD, IgE, IgG and IgM. Most of the therapeutic mabs used in the clinic belong to the IgG class, albeit other antibody classes, e.g., IgM, have been evaluated in clinical stages. Antibodies are composed of heavy chains paired with a light chain. In IgM and IgA, an additional chain, the J-chain, is present. Two types of light chains exist in humans: the κ-light chain and the λ-light chain. The κ-light chain predominates in humans and is used in the vast majority of therapeutic IgG. The reason for the preference of the κ-light chain in humans is not known. Our study investigates whether light-chain selection influences the productivity of the clinically validated mabs adalimumab and trastuzumab. Both mabs were expressed as IgG and IgM with a κ- or a λ-light chain in HEK293 cells. Besides comparing the expression levels of the different mabs, we also evaluated whether the passage number of the cell line has an impact on product yield. In addition, the expressions of adalimumab, trastuzumab, an anti-CD38 and an anti-PD-L1-antibody were analyzed in HEK293 and CHO cells when both the κ- and λ-light chains are present. In summary, IgG outperformed IgM variants in expression efficacy, while light-chain selection had minimal impact on the overall expression levels. The yields of all mab variants were higher in fresh cells, despite cell cultures with a high cell passage number having higher cell densities and cell numbers at the time of harvest. The incorporation of a particular light chain occurred at similar rates in HEK293 and CHO cells.

## 1. Introduction

The first antibody approved by the FDA was the anti-CD3 antibody muromonab, which was produced using a hybridoma cell line. Since that time, significant efforts have been made to reduce the portion of non-human protein sequences (humanization of mabs) to minimize antibody responses in patients [1]. Nowadays, antibodies are produced recombinantly, which allows for the production of molecules with fully human sequences. Therapeutic antibodies belong to the fastest growing class of new drugs and are used in the clinic for diverse therapeutic applications, such as autoimmune and cardiovascular diseases, cancer treatment and many more [2]. Antibodies are composed of two protein chains that are required for antigen binding: the heavy chain (HC) and the light chains (LC). In mammals, antibodies can be classified in five distinct classes—IgA, IgD, IgE, IgG and IgM—depending on the type of heavy chain used. In the case of IgM and IgA, a third protein chain, the joining (J)-chain, is incorporated into the architecture of the antibody. The J-chain is structural integrated within the C-terminal extensions of the IgM and IgA heavy chains and is required for the PIGR-mediated secretion of IgM and IgA [3,4]. Both the heavy and light chains are composed of a variable domain and a constant domain. The antigen-binding site, or paratope, is formed by both variable domains. In humans, there are two different light chains encoded by two different gene loci: the κ-light chain (gene locus 2p11.2) and the λ-light chain (gene locus 22q11.2) [5]. Both light-chain types consist of a variable domain and a constant domain, which determines the light-chain type. Both chains show structural and functional homologies that suggest a common evolutionary origin [6,7].

Antibody-producing cells originate from white blood cells known as B lymphocytes or B cells. Upon stimulation with a corresponding antigen, these B cells differentiate into antibody producing cells called plasma cells. The first antibody type produced by the immune system is IgM, which can be changed to another class during the immune response through a process known as class switching. During this process, the genes for the constant domains of the heavy chains are rearranged in a recombinational manner [8]. However, B cells only express one type of light chain, either the κ-light chain or the λ-light chain, but not both. The exact mechanism for the selection of the light-chain type is not known, but it is assumed that the λ-genes are used only after unsuccessful κ-light-chain recombination [9,10]. Interestingly, the ratio of κ/λ-light chains used in mature antibodies differs between different species. For example, in dogs, cats, horses and cattle, the λ-light chain is used at 90% or more [11], whereas in mice, the ratio of κ/λ-light chains is ~95%/5% [12,13]. In humans, the κ-light chain is the predominant form, and the κ/λ ratio has been reported to be ~60%/40% [14,15]. The reason for this discrepancy between different species is still unknown.

Interestingly, most of the clinically validated antibodies are those containing a κ-light chain. Referring to the data provided by the antibody society, there are 228 antibody products approved or under regulatory review. Of these, 179 antibodies contain only the κ-light chain, 26 antibodies contain only the λ-light chain, 5 antibodies have a λ- as well as a κ-light chain, and 2 products are mixtures of 2 antibodies, wherein 1 antibody contains the κ-light chain and the other the λ-light chain and 1 antibody has a light chain containing the variable domain from the λ-light chain and the constant domain from the κ-light chain [16]. It is frequently stated that usage of the λ-light chain has disadvantages during antibody developability, e.g., a higher propensity for aggregation [17]. Further reasons why λ-light-chain-containing antibodies are under-represented in the clinic may be that many antibodies have been generated in mice, which have a 99%/5% ratio of κ- to λ-light chains; therefore, the incorporation of the λ-light chain might not have been evaluated as an alternative during further antibody development. Additionally, most in vitro antibody engineering strategies are based on antibodies using the κ-light chain. In nature, the usage of the λ-light chain is probably not determined by chance. It has been reported that more complex antigens, such as influenza, led to an antibody response with a higher λ-to-κ-light-chain ratio compared to an antibody response against the diphtheria toxin [18]. Moreover, in a recent report, it was described that the yields of IgM can be increased tremendously by the incorporation of a λ-light chain instead of a κ-light chain [19]. To better understand the impact of the light-chain type on productivity, we compared the production of adalimumab and trastuzumab as IgG and IgM with either the κ- or the λ-light chain in HEK293 cells.

During the early research phase, most antibodies are produced in HEK293 cells, since HEK293 cells can be easily transfected transiently, leading to the production of sufficient amounts of protein to be used in initial screening assays [20]. Usually, there is a continuous cell culture ongoing, which is renewed in a regularly manner, e.g., after 30–40 passages. We wondered whether such frequent renewal of a cell line is actually required during the early research phase. To our knowledge, a comparable analysis of expression yields for antibodies comparing a young cell line (<15 passages) with an old cell line (>250 passages) has not been published. We have continued culturing HEK293 cells for >250 passages and used this cell line in direct comparison to a young cell line for the production of two clinically validated antibodies: adalimumab and trastuzumab

During development, the cell line is usually switched to CHO cells, where stable cell lines can be generated that can produce >10 g/L in large stirred bioreactors [21]. As reported previously, CHO cells preferably incorporate the κ-light chain over the λ-light chain when both genes are supplied. This is in contrast to HEK293 cells, for which no preference of a particular light-chain type was reported [22]. To obtain a deeper understanding of the preference of HEK293 or CHO cells for either the κ- or the λ-light chain, we selected two clinically tested IgG antibodies with either a κ-(adalimumab [anti-TNFα] and trastuzumab [anti-Erb2]) or λ-(anti-CD38- and anti-PD-L1-antibody) light chain and produced them in both cell lines, offering both light-chain genes for expression.

## 2. Materials and Methods

### 2.1. Antibody Constructs, Protein Expression and Purification

Sequences of the variable domains for the used antibodies, adalimumab and trastuzumab, were obtained from the DrugBank (https://go.drugbank.com/; trastuzumab: DB00072; adalimumab: DB0051, accessed on 15 July 2024). The Fc sequences and sequences for the constant domains of the light chains were obtained from the UniProt database (https://www.uniprot.org/; IgG1: P01857-1; IgM: P01871-1; J-chain: P01591; κ-light chain: P01834; λ-light chain: P0DOY2). The corresponding DNA was synthesized (Thermo Fisher Scientific, Waltham, MA, USA) and cloned into an expression vector under a CMW promoter and a leader sequence directing the proteins into the culture supernatant. A corresponding expression construct was made for the J-chain comprising amino acids 23-159. Plasmids were used in equimolar ratios for all transfections.

HEK293 cells (FreeStyle HEK293-F, Thermo Fisher Scientific) were cultured in suspension in 600 mL TubeSpin bioreactors (TPP Techno Plastic Products AG, Trasadingen, Switzerland) with 300 mL of FreeStyle F17 medium (Thermo Fisher Scientific) supplemented with 6 mM glutamine and 0.02% Kolliphor P180 (Sigma-Aldrich, Darmstadt, Germany) at 150 rpm, 37 °C and 8% CO_2_. For maintenance of the main cell cultures, cells were passaged 3 times per week. For cell cultures used for transfection, the cell density was ~1.5 × 10^6^ at the time of transfection, as measured with an automated cell counter (Vi-CELL BLU, Beckman Coulter, Krefeld, Germany). DNA was mixed with linear polyethylenimine (PEI, Polysciences, Warrington, PA, USA) in a ratio of 1:3 in FreeStyle F17 medium (Thermo Fisher Scientific). After incubations of 20 min at room temperature, the transfection mixtures were added to the cell cultures, and cultivation was continued for 6 days. Cells were harvested by centrifugation (2000× *g*, 20 °C, 30 min), and supernatants were 0.22 µm filtered (RapidFlow Filter Units, 0.2 µM PES Membrane, Thermo Fisher Scientific). An internal CHO-K1-derived cell line was used for protein expression. Cells were cultured in suspension in 600 mL TubeSpin bioreactors (TPP Techno Plastic Products AG, Trasadingen, Switzerland), with 250 mL of FreeStyle F17 medium, with supplements and conditions as used for HEK293 cells. For maintenance of the main cell cultures, cells were passaged 3 times per week. For cell cultures used for transfection, the cell density was ~4 × 10^6^ after centrifugation, and the medium was exchanged to new FreeStyle F17 medium with supplements. DNA was added with linear polyethylenimine in a ~ ratio of 4:6 (260 µg DNA, 440 µg sheared salmon sperm DNA [Thermo Fisher Scientific]/1000 µg PEI). After 2 h, 15% CHO CD EfficientFeed B (Thermo Fisher Scientific) was added. After transfection, cells were cultivated for 10 days. Cells were harvested as described above.

IgG proteins were purified using a protein A MabSelect Sure column (Cytiva, Freiburg, Germany) followed by a desalting step and size-exclusion chromatography (Superdex 200 20/60 pg column, Cytiva), as described before [23]. Running buffer for the size-exclusion chromatography was 10 mM Histidine and 150 mM NaCl at pH 6.0 (PAN-Biotech, Aidenbach, Germany). All experiments were conducted in duplicate or triplicate.

### 2.2. MS-Analysis

The compositions of the purified antibody preparations were analyzed by LC-MS. Protein samples were diluted to 0.25 mg/mL with water (LC-MS grade, Fisher Scientific). For glycosylation, 1.0 µL of PNGase F (#P0705L, New England Biolabs, Frankfurt am Main, Germany) was added to 25 µL of sample, and the samples were incubated at 37 °C overnight. LC-MS analysis was carried out with an 6545XT advance Bio QTOF/LC-MS system (Agilent Technologies, Santa Clara, CA, USA). For the reverse-phase HPLC, a PLRP-S column was used (Agilent Technologies, #PL1912-1502). In total, 2 µL of sample was injected and eluted with an increasing acetonitrile concentration. The eluents used were buffer A, containing LC-MS-grade water and 0.1 formic acid, and buffer B, containing 90% acetonitrile, 10% LC water and 0.1 formic acid. The obtained mass data were analyzed with MassHunter software (Agilent Technologies, V B9044.0).

### 2.3. Capillary Gel Electrophoresis (cGE)

For capillary gel electrophoresis, protein samples were diluted to a concentration of 0.5 mg/mL and analyzed on a LabChip GXII Touch (Perkin Elmer, Waltham, MA, USA). Samples were analyzed under reducing conditions (50 mM DTT) following the manufacturer’s instructions using the ProteinClear HR reagent kit (#CLS960014) and a Protein Clear HR LabChip Touch (#CLS148695, Revvity Inc., Waltham, MA, USA).

### 2.4. SDS-PAGE Analysis

Protein samples were mixed with either 4 × LDS sample buffer (Thermo Fisher Scietific) for non-reducing SDS-PAGE or with 4 × LDS sample buffer with 100 mM dithiothreitol for reducing SDS-PAGE. For SDS-PAGE assays under reducing conditions, samples were incubated for 5 min at 99 °C before loading on the gels. Here, 4–12% BisTris or 12% BisTris gels were used with MES or MOPS as running buffer (Life Technologies/Thermo Fisher Scientific). SDS-PAGE gels were stained with Coomassie blue (Instant Blue, Expedeon, Cambridgeshire, UK).

## 3. Results

**Figure 1:**
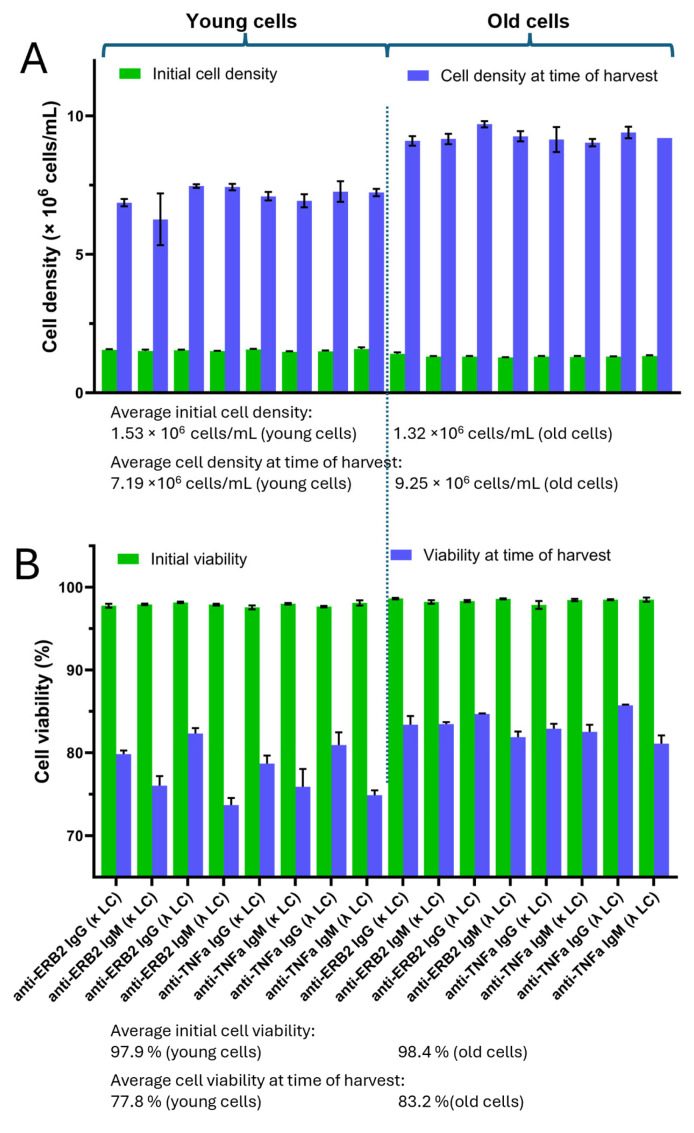
Cell densities and cell viabilities of young and old cell cultures. (**A**) At the time of transfection, all cell cultures had comparable cell densities (green bars). Cells were further incubated for 6 days to allow for protein expression. At the time of harvest, cell densities were measured again (blue bars). (**B**) At the time of transfection, the cell viability was comparable for all cell cultures (green bar). At the time of harvest, the cell viability of the young cells was more dependent on the expressed antibody type. Cell cultures expressing IgM variants had lower viability compared to cell cultures expressing IgG molecules. Cell viabilities for the old cells were slightly higher without an evident influence of the expressed antibody isoform (blue bars).

To address the preference of the light-chain type for either IgG or IgM, we produced both antibody types as variants of the anti-TNFα-antibody adalimumab and anti-Erb2 antibody trastuzumab with either the κ- or λ-light chain. All protein expression experiments were carried out using transiently transfected HEK293 cells. Concomitantly, we compared the expressions of these in antibodies in “young cells” (<15 passages) with their expressions in “old cells” (>250 passages). Young cells were transfected after 10 passages in culture after cells were withdrawn from the cell bank, where they were stored in liquid nitrogen. The “old cells” had been cultured in suspension for at least 250 passages. All cell cultures were transfected under the same conditions, having cell densities of ~1.3–1.5 × 10^6^ cells/mL and a cell viability >95% (Figure 1A). It should be noted that all cell cultures were cultivated at the same time. Before cell harvest, the cell density and viability were measured again. For the young cells, cell densities ranged between 6.8 and 7.5 × 10^6^ cells/mL. In contrast, the old cells had cell densities in the range of 9.1–9.7 × 10^6^ cells/mL (Figure 1A). The cell viability of the young cells at the time of harvest was between ~75 and 81%, whereas the cell viability of the old cells was in the range between 81 and 86% (Figure 1B). We observed that the viability of the young cells producing IgG at the time of harvest was slightly higher compared to the IgM-producing cultures (Figure 1B). Interestingly, this seems not to be the case for the old cells. In addition, the production of κ- or λ-light-chain-containing antibody variants showed no significant differences in respect to cell density and viability at the time of harvest.

**Figure 2:**
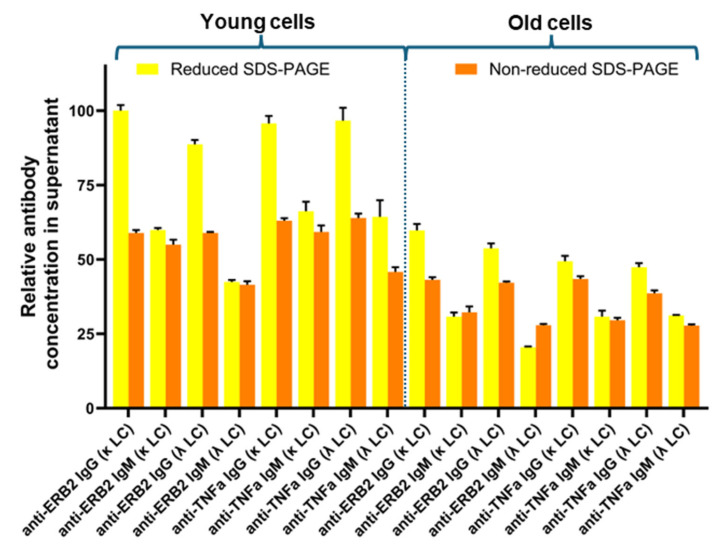
Relative antibody concentration in cell culture supernatants at the time of harvest. Protein concentrations were estimated based on the staining intensities of the SDS-PAGE gels at the time of harvest (Figures S1 and S2). In addition to the intact antibodies, excess free light chains were also secreted in the culture supernatant. Thus, the calculated amounts from the reduced SDS-PAGE gels are higher, since the excess free light chains are also considered. The expression level obtained from the reduced SDS-PAGE of anti-Erb2-IgG1(κ) was set as 100%. In general, the expression levels were higher for the young cells compared to the old cells. A clear preference for either the κ- or λ-light chain in combination with IgG or IgM cannot be inferred.

After cultivation, cells were harvested, and the culture supernatants were analyzed by SDS-PAGE (Figure S1). The expressed antibodies were clearly visible as distinct protein bands. In all cultures, excessive production of light chains is also clearly visible. As estimated from the SDS-PAGE, the amount of free light chains is higher for the κ-light chain compared to the λ-light chain (Figure S1). To compare the expression levels of the different antibody variants, the staining intensity of the bands in both the reduced and non-reduced SDS-PAGE gels was measured, and the value measured for anti-Erb2-IgG (κ) was arbitrarily set as 100%. For the reduced-SDS-PAGE gels, the staining intensities of both the heavy and light chains were considered (Figure S2, Table S1). The relative expression data are shown in Figure 2. Based on the reduced SDS-PAGE gels, the expression yields are higher for the IgG variants compared to the IgM variants. This observation is more evident for the young cells compared to the old cells. The reason for this is probably that in the calculation for the reduced antibodies, the amount of free light chain is also considered. For the IgG variants, the usage of either the κ- or the λ-light chain does not have a big impact on the expression yield. For the IgM variants, the yields seem to be slightly lower when the λ-light chain is used. However, this effect is even smaller for the anti-TNFα-IgM variant compared to the anti-Erb2-IgM variant. In the case of the old cells, there is no difference in expression levels between anti-TNFα-IgM(κ) and anti-TNFα-IgM(λ). As judged from the SDS-PAGE (Figure S1), free κ-light chains are secreted in higher amounts compared to free λ-light chains, but excess light-chain expression does not seem to have an impact on overall antibody expression. Probably, the rate limiting step in antibody secretion is the assembly of the light chains with the heavy chains in the endoplasmic reticulum. In general, the expression levels are generally lower for the older cells compared to the fresh cells.

**Figure 3:**
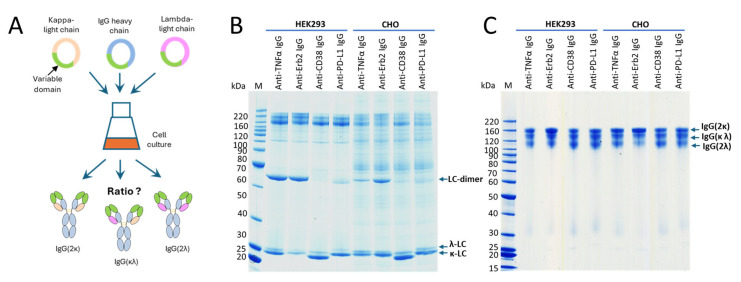
Incorporation of a κ- versus a λ-light chain during co-expression. (**A**) Schematic representation of the co-expression experiment. For all antibody variants, cells were transiently transfected with plasmids coding for the κ- and λ-light chains as well as the heavy chain in a 1:1:1 ratio. (**B**) SDS-PAGE from the culture supernatants at the time of harvest. The protein bands corresponding to the different light-chain isotypes as well as the light-chain (LC) dimer are indicated (12% BisTris gel). (**C**) SDS-PAGE of purified proteins (4–12% BisTris gel). The chosen running conditions allow for the separation of the different antibody species, which are indicated correspondingly. All SDS-PAGE experiments were run under non-reducing conditions using MOPS as the running buffer.

**Figure 4:**
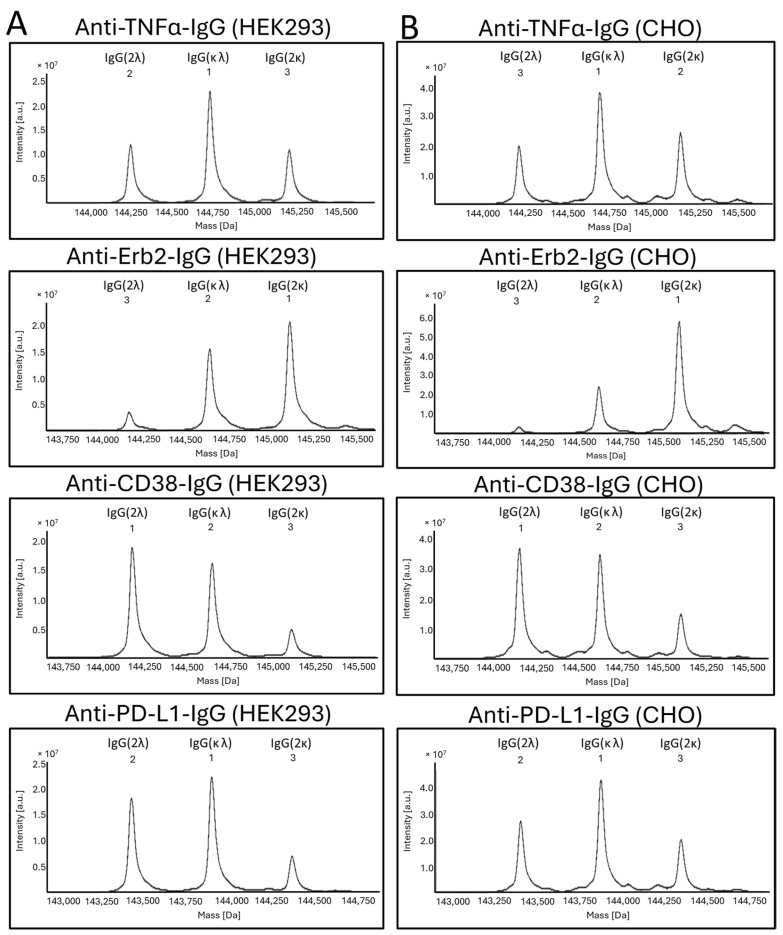
Mass spectrometry spectra of purified antibodies. All measurements were conducted after deglycosylation and under non-reducing conditions. The signals are numerated according to the signal intensities. The antibody species corresponding to each signal are indicated. (**A**) MS spectra of antibodies produced in HEK293 cells. (**B**) MS spectra of antibodies produced in CHO cells. The corresponding signal intensities are given in Table 1.

**Figure 5:**
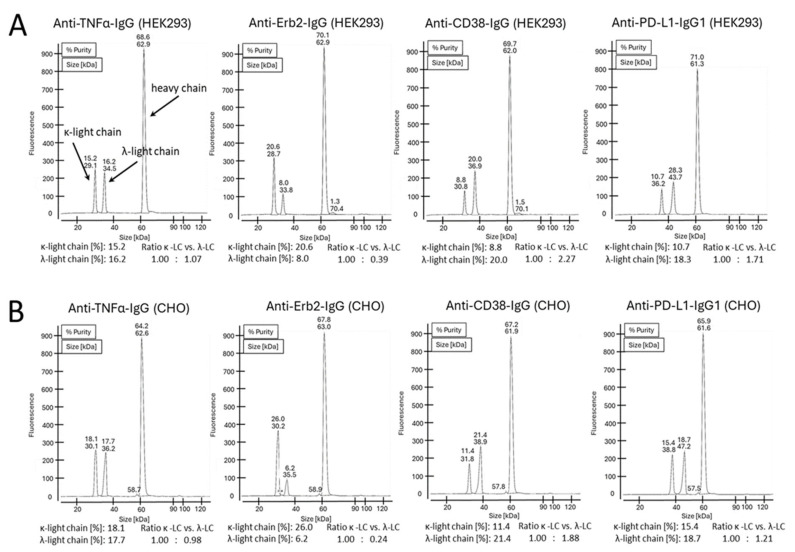
cGE chromatograms of purified antibodies. All measurements were conducted with reduced samples. The signals corresponding to the κ-and λ-light chains as well as heavy chain are indicated. (**A**) cGE chromatograms of antibodies produced in HEK293 cells. (**B**) cGE chromatograms of antibodies produced in CHO cells. The calculated area of the κ- and λ-light-chain signals are highlighted. Based on these values, the ratio of total κ- vs. λ-light chain that were incorporated into the IgG molecule was calculated.

It has been reported earlier that CHO cells have a strong preference for the incorporation of the κ-light chain over the λ-light chain. For HEK293 cells, no preference for either light-chain isotype was reported [22]. To further address the question whether a certain light-chain isoform is preferred over the other one, we expressed four clinically validated antibodies as IgG offering both the κ- and the λ-light chain genes for expression simultaneously. The antibodies with an original κ-light chain are an anti-TNFα- and an anti-Erb2-IgG, and the antibodies with an original λ-light chain are an anti-CD38- and an anti-PD-L1-IgG. HEK293 and CHO cells were transiently transfected using the same plasmids, with three plasmids in a 1:1:1 ratio encoding for the heavy chain and a κ- and a λ-light chain (Figure 3A). At the time of harvest, samples from the cell culture supernatants were analyzed by SDS-PAGE (Figure 3B). As shown, both light chains were highly expressed, and free light chains were secreted. As estimated from the staining intensities of the bands in SDS-PAGE, secretion levels are higher for the free κ-light chain compared to the free λ-light chain. Interestingly, the amount of light-chain dimer formation is different between the different antibodies. High amounts of light-chain dimers are clearly visible for adalimumab and trastuzumab (originally with the κ-light chain), but only small amounts are observed for the anti-CD38- and anti-PD-L1-IgG (originally with the λ-light chain). The results obtained for excess light-chain expression from HEK293 and CHO cells are comparable (Figure 3B). These observations are in accordance with the results obtained in the previous experiment. SDS-PAGE analysis was also conducted with the purified proteins under reducing and non-reducing conditions using two different SDS-PAGE running conditions. Using MES as the running buffer, separation of the different species could not be observed (Figure S3). In contrast, when using a MOPS-based running buffer, a clear separation of the different antibody species could be observed (Figure 3C). The separation is obviously due to the different isoelectric points of the antibodies having two κ-, two λ- or one κ- and one λ-light chain. Under reducing conditions, distinct bands are clearly visible for the heavy and light chains. However, no separation of the κ- and the λ-light chains was observed in the SDS-PAGE gels of the purified IgG antibodies under reducing conditions (Figure S3B,C), despite this being clearly visible in the SDS-PAGE gels of the culture supernatants under non-reducing conditions (Figure 3B). To estimate the ratio between the different antibody species, the staining intensities of the corresponding bands from the SDS-PAGE were measured (Figure S4). We noticed different amounts of secreted free κ- or λ-light chain for the different IgG variants. These differences do not translate into the corresponding incorporation rates of the light chains into the antibodies. Reasons for this are unknown but are obviously related to the antibody assembly within the endoplasmic reticulum (ER). Purified proteins were also analyzed by mass spectrometry (Figure 4). No differences in light-chain incorporation between antibodies expressed in HEK293 or CHO cells could be observed. The obtained masses were in good accordance with the calculated masses (Table S2). The results from the volumetric analysis of the stained SDS-PAGE gels and signal intensities obtained by MS are given in Table 1. Despite that the signal intensities obtained by MS do not necessarily allow for a quantification of the analyzed species, the obtained data from the two different methods are in good accordance. All antibodies were also analyzed under reducing conditions by cGE, where a good separation of the κ- and λ-light chains was achieved. Based on the obtained signal intensities, the ratio of incorporated κ- vs. λ-light chains into an IgG was calculated (Figure 5). Data from cGE are in accordance with data from MS and SDS-PAGE.

Taken together, in the case of anti-TNFα-IgG, HEK293 cells obviously do not discriminate between κ- and λ-light chains. Both light chains are used in equal amounts. A slight preference for the usage of the κ-light chain is observed for CHO cells compared to HEK293 cells. In contrast to anti-TNFα-IgG, for the anti-Erb2-IgG, the incorporation of a κ-light chain is clearly preferred by both HEK293 and CHO cells. Again, the CHO cells had a slightly higher incorporation rate for the κ-light chain compared to HEK293 cells. For the anti-CD38-IgG, the incorporation of the λ-light chain is clearly preferred in both cell types. Again, CHO cells incorporated more κ-light chain compared to HEK293 cells. Similar results as for the anti-CD38-IgG were obtained for the anti-PD-L1-IgG, albeit for the latter, a slightly higher percentage of κ-light chain was incorporated. There is a perceptible trend within the data. Overall, CHO cells actually seem to prefer the κ-light chain over the λ-light chain compared to HEK293 cells, but this extent is rather small. However, it is evident that the selection and incorporation of a particular light-chain isotype does not obviously depend on the host cell type but rather on the original antibody isoform.

## 4. Discussion

In the first part of our study, we compared the expressions of two validated monoclonal antibodies, adalimumab and trastuzmab, in different antibody isoforms, IgM and IgG, and analyzed whether the usage of either the κ- or λ-light chain had a beneficial effect on the expression yields. These experiments were carried out using transient transfection of the HEK293 cell line having different passages (<15 young cells, >250 old cells). All expression experiments were conducted at the same time under the same conditions to allow for the best comparison between the experiments. All cell cultures had comparable initial cell densities and viabilities (Figure 1A). We observed that at the time of harvest, the cell densities and viabilities of cultures with old cells were higher than cell cultures with young cells. Within the young cells, the viabilities were higher for cells expressing IgG compared to IgM. This bias was not observed for the old cells. We do not know the reason for this, but we assume that the assembly of IgM molecules, consisting of 3 different and, in total, 21 protein chains, triggers ER stress, leading to reduced viability. During the “aging” period, cells have obviously adapted to cell growth and not for protein synthesis. In the case of the assembly of large protein complexes, reduced protein synthesis also leads to reduced ER stress and, hence, enhanced viability. It should be noted that during the maturation of B lymphocytes into IgM-producing plasma cells, specific chaperones are expressed to facilitate IgM synthesis [24,25]. The older cells have evolved into a fast-growing culture rather than a good production cell line, as evident from the reduced expression levels compared to the young cells (Figure 2). The underlying changes for these observations are unknown, as we did not conduct genomic or transcriptional profiling of the cell lines. However, when aiming at high expression levels, it is expedient to use fresh cells. It is well known that cells can adapt to their environment by altering their genomic and transcriptomic profiles. A well-characterized example is the evolution of the parental HEK293 cell line and its derivatives [26]. In contrast to the HEK293 cell lines analyzed by Malm et al. [26], which have evolved over several years or decades, the changes observed with our cells are evident after 250 passages (3 passages/week, ~1.6 years) without any further selection pressure.

Next, we evaluated the expression levels of two IgM variants and two IgG antibodies with the κ- as well as the λ-light chains. As it has been reported earlier, the usage of the λ-light chain for IgM expression instead of the κ-light chain should have a huge beneficial effect on the expression yields. Gong et al. evaluated the expressions of four antibodies as IgG, IgA and IgM with either the κ- or λ-light chain. Their observation was that IgM variants with a κ-light chain showed only very little expression. Upon usage of the λ-light chain instead of the κ-light chain, the expression levels for one IgM variant improved by a factor of 12 (VRC01). For the other IgM variants, an increase in the expression levels of ~7000-fold (PGT121), ~11.600-fold (33C6) and even up to nearly ~20.000-fold (Fm-6) was described [19]. Despite having only a limited data set, we could not observe a meaningful change in the expression levels for the IgM antibodies when using the λ-light chain instead of the κ-light chain. A possible explanation for the large discrepancy between the expression levels of IgM molecules with either the κ- or λ-light chain, as described by Gong et al. and our results, might be that Gong et al. directly analyzed culture supernatants of the antibody-expressing cells. Analyses were carried out using ELISAs directed against the κ- or λ-light chain [19]. This experimental setup does not allow us to discriminate between the detection of light chains incorporated into an antibody or free light chains.

During the early research phase, monoclonal antibodies are usually produced in transiently transfected HEK293 cells. This cell line is derived from human embryonic kidney cells and used because of its ease of transformation and high expression titers [20]. For the development and production of monoclonal antibodies, the expression host is usually switched to CHO cells, which are derived from the ovary of the Chinese hamster *Cricetulus griseus*. CHO cells can easily be adapted for growth in the large stirred bioreactors required for the large-scale production of biotherapeutics with high expression titers of <10 g/L [21]. Most of the clinically validated IgG variants have a κ-light chain, as antibodies with a λ-light chain may have a tendency for aggregation [17]. Hence, the light-chain type chosen for production is usually the κ-light chain. Further support for using the κ-light chain for CHO cell expression was presented in a recent report by Zhou et al., wherein the preferred incorporation of the κ-light chain over the λ-light chain was described for CHO cells but not for HEK293 cells [22]. Upon co-transfection of CHO and HEK293 cells with a heavy chain and the κ- and λ-light chains, a 99:1 κ/λ-light-chain usage was reported for CHO cells, as well as a ~1:1 ratio for HEK293 cells [22]. To further understand the impact of the κ-or the λ-light chain, we chose two validated IgG antibodies with a κ-light chain and two IgG variants with a λ-light chain and transiently transfected either CHO or HEK293 cells with three plasmids coding for the heavy chain and the κ- and the λ-light chains in a 1:1:1 ration. (Figure 3). The purified IgG variants were analyzed by SDS-PAGE, cGE and MS. We observed only slight differences in the usage of either light-chain isotype between HEK293 and CHO cells. Interestingly, we noticed that the originally used light-chain isotype is the preferred one in both cell lines. This effect is largely dependent on the particular antibody used. In the case for the anti-TNFα antibody adalimumab, the light-chain isoform does not have an influence on incorporation in the IgG. In contrast, for the anti-Erb2 antibody trastuzumab, the usage of the κ-light chain is clearly preferred over the λ-light chain in both cell lines to similar extents. This phenomenon was also observed for the two antibodies naturally having a λ-light chain. The trend for using the λ-light chain is more pronounced for the anti-CD38 antibody compared to the anti-PD1 antibody. Overall, there seems to be a slightly higher usage of the λ-light chain in CHO cells compared to HEK293 cells, but the most important factor for selecting the best light-chain isotype seems to be the usage of the original light-chain isotype that was selected by the antibody-producing B cell. This is an important factor to be considered when switching the light-chain type from the λ- to the κ-isotype. Possibly, additional engineering efforts might be required.

In summary, we addressed several topics that frequently arise during the early research phase for antibodies. HEK293 cells quickly adapt to culture conditions; in our case older cells were growing faster but produced proteins in lower yields. We did not observe a positive effect upon the usage of the λ-light chain for enhanced expression for IgM antibodies. Furthermore, only a very slight preference of CHO cells for the κ- over the λ-light chain was observed. In conclusion, the light-chain isotype that was originally selected by the antibody-producing B cell is a critical factor to be considered for therapeutic antibody generation.

## Figures and Tables

**Table 1:** Calculated ratios between antibodies with different light-chain compositions expressed in HEK293 and CHO cells as measured by MS (Figure 4) and obtained from non-reduced SDS-PAGE (Figure 3C).

		HEK293 Expression	CHO Expression	HEK293 Expression	CHO Expression
		Non-Reduced SDS-PAGE		MS (Signal Intensity)	
Parental mab	Isoform	[%]	[%]	[%]	[%]
Anti-TNFα-IgG	IgG(2κ)	35.7	36.4	23.9	29.7
	IgG(2λ)	31.6	30.4	26.1	24.3
	IgG(κλ)	32.7	33.2	50.0	46.1
Anti-Erb2-IgG	IgG(2κ)	46.4	50.3	52.0	67.4
	IgG(2λ)	25.0	19.0	8.8	4.1
	IgG(κλ)	28.6	30.7	39.2	28.5
Anti-CD38-IgG	IgG(2κ)	28.2	27.3	12.1	17.2
	IgG(2λ)	37.5	38.2	47.3	42.5
	IgG(κλ)	34.3	34.5	40.6	40.2
Anti-PD-L1-IgG	IgG(2κ)	28.9	31.8	14.8	22.3
	IgG(2λ)	36.1	30.0	38.3	30.3
	IgG(κλ)	35.0	38.2	46.9	47.4

## Data Availability

The original contributions presented in this study are included in the article/Supplementary Materials. Further inquiries can be directed to the corresponding author.

## Supplementary Materials

The following supporting information can be downloaded at: https://www.mdpi.com/article/10.3390/antib14030053/s1, Figure S1: SDS-PAGE of the culture supernatant at the time of harvest. Figure S2: Volumetric measurement of the staining intensities of the SDS-PAGE, as shown in Figure S1. Figure S3: SDS-PAGE of purified IgG antibodies. Figure S4: Volumetric measurement of the staining intensities of the SDS-PAGE, as shown in Figure 3C. Table S1: Volumetric analysis of the SDS-PAGE from Figures S1 and S2: Table S2: Calculated masses and masses obtained by MS for each purified antibody.
