# Supplementary material for: Impact of Light-Chain Variants on the Expression of Therapeutic Monoclonal Antibodies in HEK293 and CHO Cells
Source: Antibodies (Basel). 2025 Jun 24;14(3):53. doi: 10.3390/antib14030053 (PMC12286288; doi:10.3390/antib14030053)
Supplement: Supplementary file 1 [file antibodies-14-00053-s001.zip › antibodies-3680605-supplementary.pdf]

Impact of Light Chain Variants on the Expression of Therapeutic Monoclonal Antibodies in HEK293 and CHO Cells

Alexander Veber[1,2], Dennis Lenau[1], Polyniki Gkragkopoulou[1], David Kornblüh Bauer[1], Ingo Focken[1], Wulf Dirk Leuschner[1], Christian Beil[1],Sandra Weil[1], Ercole Rao[1], Thomas Langer[1*]

[1] Sanofi-Aventis Deutschland GmbH, R&D Large Molecules Research, Industriepark Höchst, 65926 Frankfurt am Main, Germany.

[2] Provadis School of International Management and Technology AG, Industriepark Höchst, Building B845, 65926 Frankfurt am Main, Germany

*Corresponding author:

Thomas Langer (thomas.langer@sanofi.com)

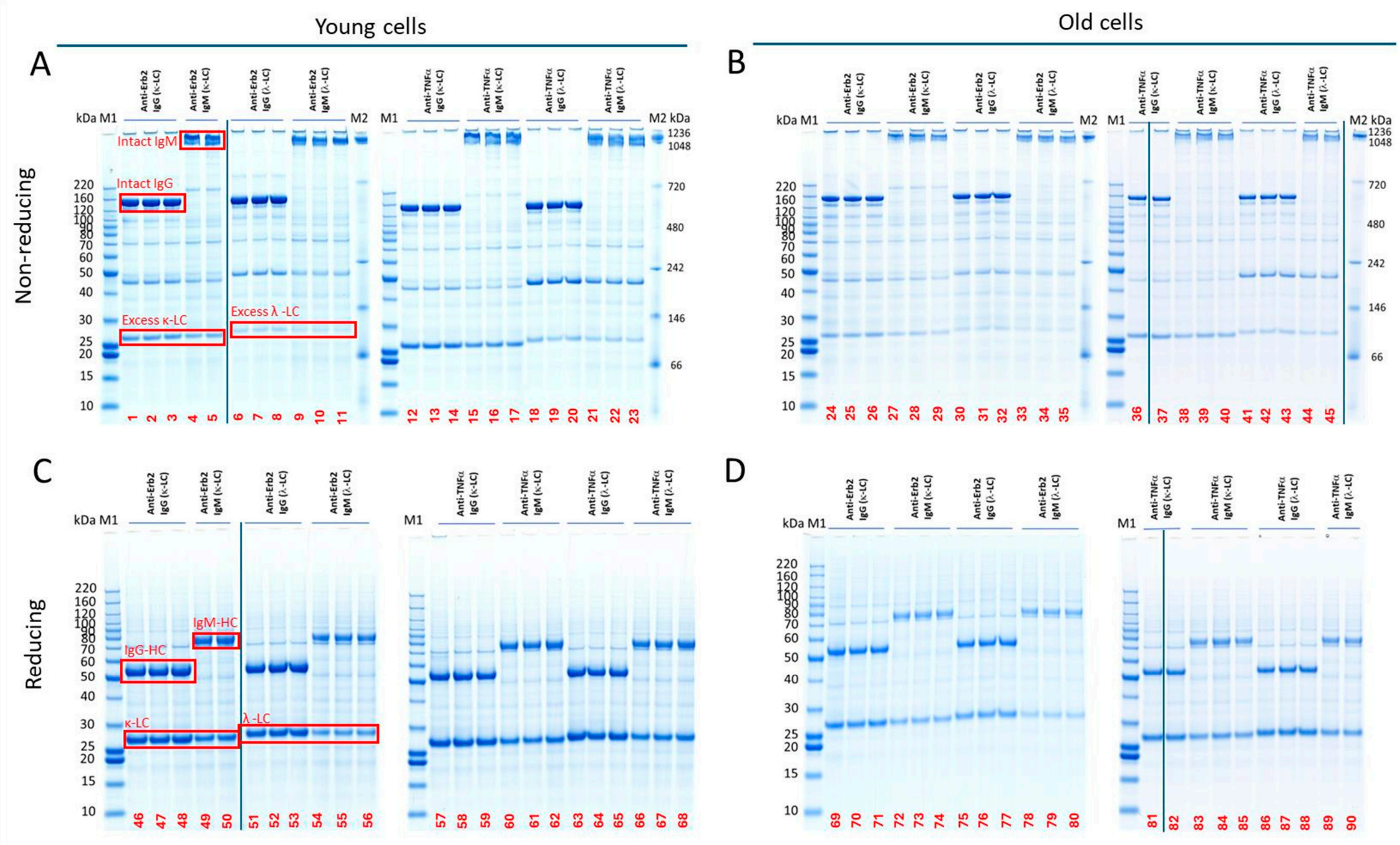

Figure S1. SDS-PAGES of the culture supernatant at the time of harvest. Proteins were analyzed under non-reducing (A, B) and reducing conditions (C, D). Under non-reducing condition the intact IgG migrates as a single band with an apparent molecular wight of approx. 160 kDa. The intact IgM antibody consist probably of a mixture of pentamers and hexamers as revealed by the double band. Excess free light chains are also visible. As estimated from the staining intensities, free κ-light chain seems to be produced in higher amounts compared to free λ-light chain. The corresponding species are exemplary labeled in (A).

Under reducing conditions, the antibodies are separated into light chain (LC) and heavy chain (HC). Both IgG and IgM heavy chains migrate as a distinct single band in the SDS-PAGE with an apparent molecular wight of ~ 55 kDa and 70 kDa, respectively. The corresponding chains are exemplary labeled in (C). Note, that the λ-light chain has a higher apparent molecular weight in the SDS-PAGE compared to the κ-light chain despite its lower molecular weight. M1: BenchMark protein ladder, M2: Native Mark (all protein markers are from Thermo Fisher Scientific). All lanes in the different SDS-PAGE are numbered consecutively for clarity with red numbers. In gels containing a black bar, non-relevant bands were cropped.

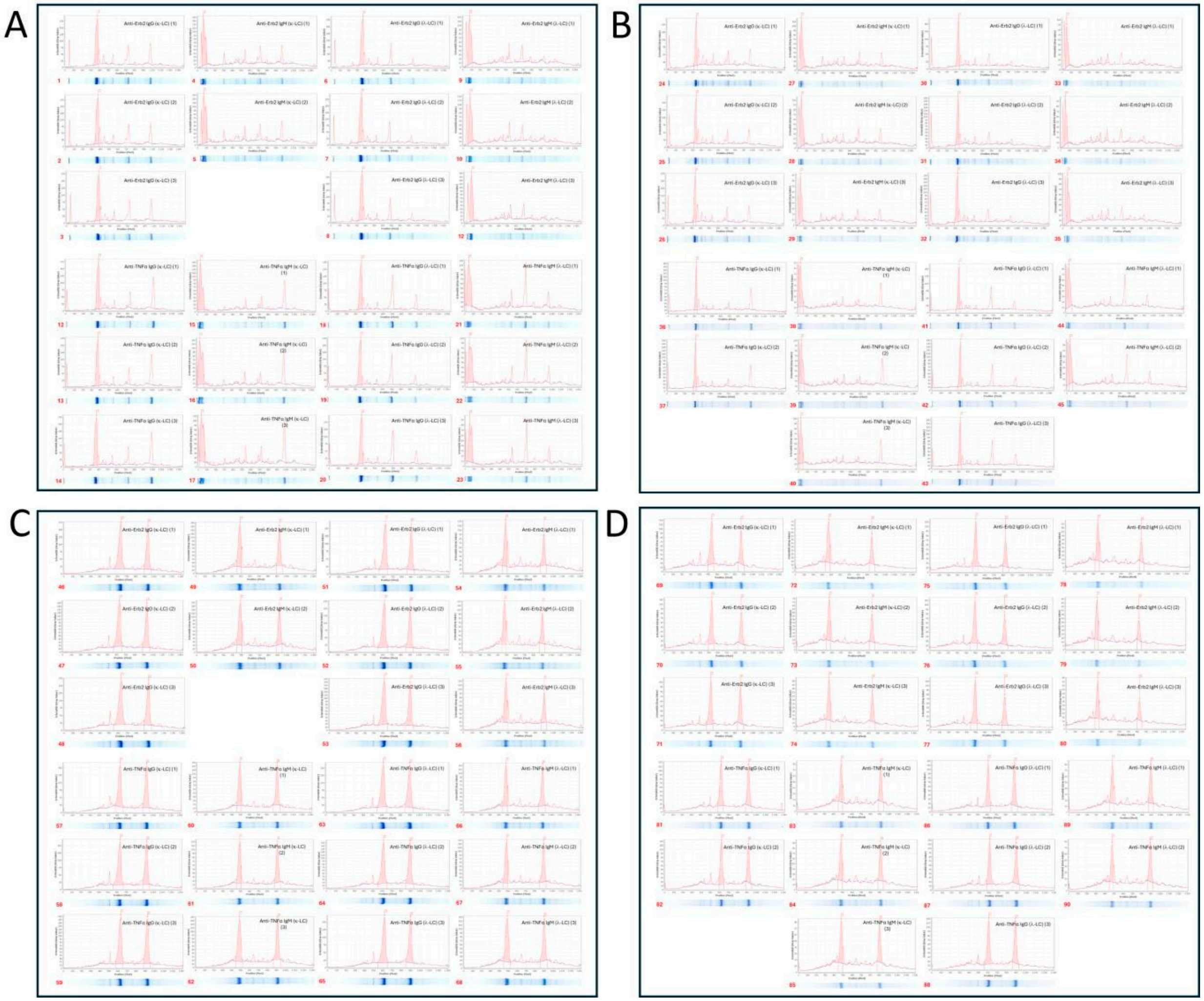

Figure S2. Volumetric measurement of the staining intensities of the SDS-PAGE as shown in Fig S1. The numbering of the single lanes corresponds to the labeling of the lanes of the SDS-PAGEs in Fig S1 (red numbers). Proteins were analyzed under non-reducing (A, B) and reducing conditions (C, D). The upper pictures show the protein lane from the SDS-PAGE in a chromatogram-style above the lane from the SDS-PAGE. The colored signals were used for calculation.

To compare the expression levels, the staining intensities of the bands from the young cells expressing the anti-Erb2-IgG (κ) was arbitrarily set as 100% for reduced and non-reduced SDS-PAGE. For the non-reduced samples, the bands corresponding to the intact antibody were used for calculation. For reduced samples, the bands corresponding to the light chain and the heavy chain were used for calculation. Image analysis was done with the program LabImage 1D V 4.5.0 (Kapelan Bio-Imaging GmbH). The staining intensities of the protein bands are given as dimensionless volume-value (Tab. S1).

Table S1. Volumetric analysis of the SDS-PAGE from FigS1, S2. The calculated volume-values for each band are shown. All sample lanes are labeled with the consecutively numbering corresponding to FigS1, S2. The values obtained from anti-Erb2 IgG were arbitrary set as 100% (non-reduced: lane 1, signal from intact IgG; reduced: lane 46 signal from both, heavy chain and light chain) (nd: no data available).

		Fig S1A (Gel 1/2)		Fig S1B (Gel 1/2)		Fig S1C (Gel 1/2)			Fig S1D (Gel 1/2)	
		Young cells , non reduced		Old cells , non reduced		Young cells , reduced			Old cells , non reduced	
		intact mab		intact mab		HC	LC		HC	LC
antibody variant	Lane	[Volume]	Lane	[Volume]	Lane	[Volume]	[Volume]	Lane	[Volume]	[Volume]
Anti-Erb2 IgG (κ-LC) (1)	1	429,57	24	279,63	46	528,83	399,92	69	263,4	174,76
Anti-Erb2 IgG (κ-LC) (2)	2	435,59	25	260,27	47	542,07	448,44	70	230,25	156,5
Anti-Erb2 IgG (κ-LC) (3)	3	411,02	26	267,86	48	550,5	418,38	71	230,61	182,03
Anti-Erb2 IgM (κ-LC) (1)	4	386,44	27	221,54	49	330,83	239,54	72	118,68	77,52
Anti-Erb2 IgM (κ-LC) (2)	5	410,05	28	205,58	50	336,55	245,64	73	126,76	84,01
Anti-Erb2 IgM (κ-LC) (3)		nd	29	177,66		nd	nd	74	134,9	94,55
Anti-Erb2 IgG (λ-LC) (1)	6	425,28	30	262,8	51	509,27	369,95	75	221,8	133,6
Anti-Erb2 IgG (λ-LC) (2)	7	421,89	31	259,07	52	493,91	345,21	76	223,11	140,33
Anti-Erb2 IgG (λ-LC) (3)	8	430,74	32	268,41	53	478,32	362,13	77	235,73	157,96
Anti-Erb2 IgM (κ-LC) (1)	9	314,5	33	177,85	54	236,23	161,12	78	93,11	53,11
Anti-Erb2 IgM (κ-LC) (2)	10	285,53	34	174,68	55	250	162,24	79	88,89	47,64
Anti-Erb2 IgM (κ-LC) (3)	11	297,58	35	168,8	56	250,51	167,54	80	92,06	49,65
Anti-TNFα IgG (κ-LC) (1)	12	378,03	36	215,95	57	448,74	427,9	81	256,31	236,81
Anti-TNFα IgG (κ-LC) (2)	13	374,23	37	208,77	58	446,53	376,36	82	247,69	211,75
Anti-TNFα IgG (κ-LC) (3)	14	360,4		nd	59	418,22	384,31		nd	nd
Anti-TNFα IgM (κ-LC) (1)	15	367,29	38	137,7	60	290,66	266,19	83	150,11	140,5
Anti-TNFα IgM (κ-LC) (2)	16	354,92	39	151,16	61	285,82	255,67	84	172,15	162,32
Anti-TNFα IgM (κ-LC) (3)	17	325,02	40	144,33	62	360,39	270,57	85	139,44	130,03

Anti-TNFα IgG (λ-LC) (1)	18	391,34	41	187,15	63	466,11	449,32	86	219,43	222,15
Anti-TNFα IgG (λ-LC) (2)	19	360,21	42	196,95	64	410,7	372,59	87	222,54	222,72
Anti-TNFα IgG (λ-LC) (3)	20	377,18	43	181,97	65	436,19	389,97	88	253,32	230,23
Anti-TNFα IgM (κ-LC) (1)	21	271,09	44	136,62	66	250,64	245,32	89	143,38	160,05
Anti-TNFα IgM (κ-LC) (2)	22	285,6	45	134,52	67	270,74	256,14	90	140,24	160,4
Anti-TNFα IgM (κ-LC) (3)	23	252,42		nd	68	366,26	288,3		nd	nd

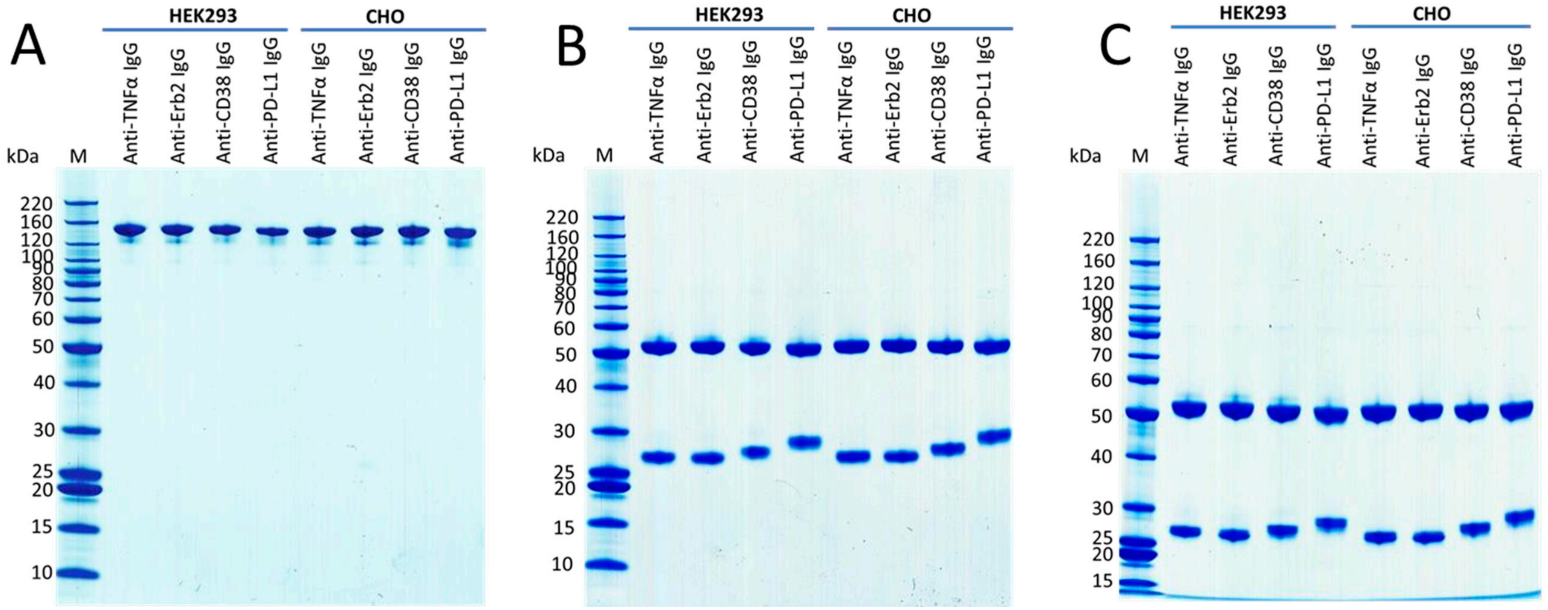

Figure S3. **SDS-PAGE of purified IgG antibodies**. The cells used for expression are indicated. A) SDS-PAGE under non-reducing conditions using a MESbuffer as running buffer. B) SDS-PAGE under reducing conditions using MES-buffer as running buffer. C) SDS-PAGE under reducing conditions using MOPSbuffer as running buffer. In contrast to the SDS-PAGE in Fig 3B, which was run under non-reducing conditions, no separation between κ- and λ-light chain occurred in SDS-PAGE running under reduced-conditions.

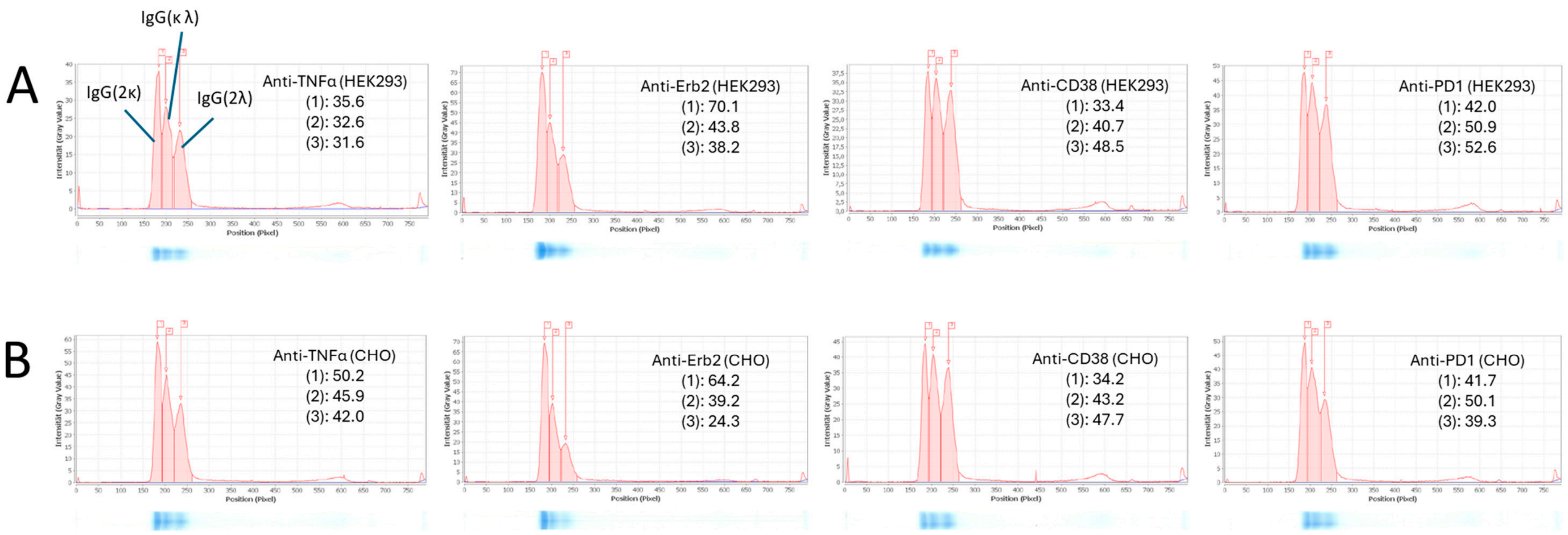

Figure S4. Volumetric measurement of the staining intensities of the SDS-PAGE as shown in Fig 3C (purified IgGs from coexpression of heavy chain, κ- and λ-light chain. A) Expression in HEK293 cells B) expression in CHO cells. The upper pictures show the protein lane from the SDS-PAGE in a chromatogram-style above the lane from the SDS-PAGE. In each chromatogram the first signal is from IgG(2κ), the second signal from IgG(κλ), and the third signal from IgG(2λ), as indicated for anti-TNFα(HEK293). Staining intensities of the protein bands are given as dimensionless volume-value. Image analysis was done with the program LabImage 1D V 4.5.0 (Kapelan Bio-Imaging GmbH).

Table S2. **Calculated masses and masses obtained by MS for each purified antibody**. Molecular masses were calculated within the GeneData Biologics database software (GeneData AG, Basel, Switzerland). All cysteines were assumed to form disulfide-bridges.

Antibody	IgG 2κ (Da)	IgG κλ (Da)	IgG 2λ (Da)
Anti-TNFα (calculated Mw)	145.189	144.718	144.246
Anti-TNFα (HEK293) (measured Mw)	145.197	144.725	144.253
Anti-TNFα (CHO) (measured Mw)	145.196	144.724	144.252
Anti-Erb2(calculated Mw)	145.101	144.630	144.158
Anti-Erb2 (HEK293) (measured Mw)	145.108	144.637	144.164
Anti-Erb2 (CHO) (measured Mw)	145.108	144.635	144.166
Anti-CD38 (calculated Mw)	145.100	144.629	144.158
Anti-CD38 (HEK293) (measured Mw)	145.107	144.636	144.164
Anti-CD38 (CHO) (measured Mw)	145.108	144.636	144.165
Anti-PD-L1 (calculated Mw)	144.374	143.903	143.431
Anti-PD-L1 (HEK293) (measured Mw)	144.382	143.910	143.438
Anti-PD-L1 (CHO) (measured Mw)	144.382	143.910	143.439
